# Visual assessment of causality in the Poisson effect

**DOI:** 10.1038/s41598-019-51509-x

**Published:** 2019-10-18

**Authors:** Takahiro Kawabe

**Affiliations:** NTT Communication Science Laboratories, Atsugi, Japan

**Keywords:** Object vision, Human behaviour

## Abstract

When a material is stretched along a spatial axis, it is causally compressed along the orthogonal axis, as quantified in the Poisson effect. The present study examined how human observers assess this causality. Stimuli were video clips of a white rectangular region that was horizontally stretched while it was vertically compressed, with spatially sinusoidal modulation of the magnitude of vertical compressions. It was found that the Poisson’s ratio—a well-defined index of the Poisson effect—was not an explanatory factor for the degree of reported causality. Instead, reported causality was explained by image features related to deformation magnitudes. Comparing a material’s shape before and after deformation was not always required for the causality assessment. This suggests that human observers determine causality in the Poisson effect by using heuristics based on image features not necessarily related to the physical properties of the material.

## Introduction

In everyday life, human observers easily discriminate such physical properties of a material as viscosity, elasticity, plasticity, and so forth. Previous studies have reported the visual information that contributed to discrimination of physical properties. For example, the assessment of liquid viscosity is clearly explained in terms of image motion characteristics^1^ and/or mid-level image features^2,3^. Human observers are also good at discriminating the stiffness of cloth such as silk and cotton. Recent studies^4,5^ showed that the observers adroitly used image motion characteristics to discriminate the cloth’s stiffness. The assessment of elasticity for jelly-like materials is likely made on the basis of image parameters such as motion and shape^6–8^. In this way, human observers make use of static and dynamic image characteristics to assess the physical properties of objects and materials in the world.

**Figure 1:**
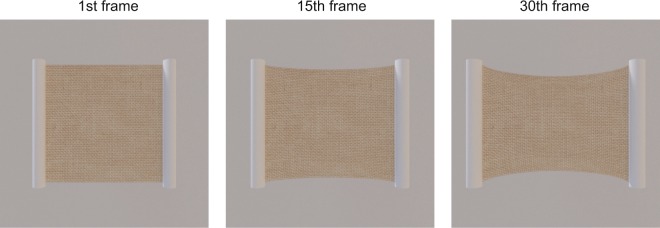
Snapshots of the video clip which simulates a horizontally stretched cloth by using Blender (https://www.blender.org/). As the cloth is horizontally extended across video frames, vertical compression of the cloth occurs.

A next important step in the research in material perception is to investigate how well human observers understand the physical behavior of materials. For example, when an extension force is applied to an elastic material along a single spatial axis, the material is at the same time compressed along the orthogonal axis (Fig. 1). This physical phenomenon is called the Poisson effect. Usually, the magnitude of compression is smaller than the magnitude of extension. The size of the Poisson effect is described by Poisson’s ratio *ν*, which is derived from the following formula,

1 $$\nu =|\frac{{d}_{y}/h}{{d}_{x}/w}|$$

where *w* is the length of the material along its axis of extension, *h* is the length of the material along its compression axis, *d*_*x*_ is the magnitude of extension and *d*_*y*_ is the magnitude of compression. The value of *ν* occupies the range below 0.5, depending on the type of material. For example, the Poisson ratio of cork is 0.0, the ratio of metal is 0.3 and the ratio of natural rubber is 0.5.

In our daily life, human observers do not feel the Poisson effect is an unusual phenomenon. For example, it is common to experience that when we stretch fabric horizontally, the fabric is vertically compressed. To date, however, it has remained unclear how human observers combined the horizontal extension and vertical compression in the Poisson effect to judge the apparent causality of such deformations.

There are two possible strategies used by human observers to assess causality in the deformation. One strategy would be to directly represent the Poisson effect by generating a representation of the physical properties of elastic materials. Previous studies have shown that human observers anticipated the future of physical events on the basis of internalized physics (i.e., naive physics^9,10^) or an internalized physics engine that is conceptually similar to the physics engines used in computer graphics^11,12^. Thus, there was a possibility that observers might internally represent Poisson’s ratio and use the ratio to assess the causality of deformation. Generally, the proportion of reports of no causality would increase with Poisson’s ratio. Critically, it was expected that if human observers judged the causality of deformation in terms of a critical value of Poisson’s ratio (i.e., threshold Poisson’s ratio), the threshold Poisson’s ratio would be constant across initial widths of stimuli because Poisson’s ratio was determined as a function of initial widths as well as compression magnitudes, as described in Formula 1, and human observers would have base their judgments of causality on the same formulation of the internally represented Poisson’s effect. I did not make a specific prediction about the relationship between specific values of Poisson’s ratio and the observers’ responses because at this stage it was unclear whether human observers actually internalized Poisson’s ratio.

The other strategy would be to take advantage of image features in assessing causality. Recent studies in material perception suggest that the visual system uses heuristics based on mid-level visual features to infer material properties^13–15^. In particular, as described above, some studies have already provided evidence that human observers use dynamic and static image features to assess the elasticity of materials^4–8^. Thus, in addition to material perception, the causality judgment may also be based on the image features related to the extension and/or compression of materials. Here no assumption is made that the causality judgment of deformations should follow the perception of elastic materials. Instead, I assume that human observers assess the causality of deformations in the Poisson effect in parallel with the determination of material types. Because there are many types of materials that show the Poisson effect, it may not be a good strategy for human observers to determine the material type in advance of the casualty judgment of deformation.

In this study the focus was on the ratio of the area of deformed regions to the area of original regions of the material, as an index of deformation magnitude (Fig. 1b). It is known that image area is an important visual cue used to assess liquid viscosity from images^2,3^. The degree of material deformation in the stimulus clip was quantified by calculating the ratio of the deformed area to the area of the original, intact material. The area ratio increased with the magnitude of image deformation. It was expected that the reports of no causality would increase with the area ratio.

The purpose of Experiment 1 was to explore, without a strong hypothesis, whether Poisson’s ratio or the area ratio could account for the causality perception of the Poisson effect in a systematic way. In Experiment 2, the role of the area ratio in the determination of causality was directly assessed by manipulating stimulus durations that could affect the area ratio. Experiment 3 checked whether contour shape played a critical role in the assessment of causality. Overall, the results suggest that human observers use image features (that is, the area ratio which is related to deformation magnitudes) in order to determine causality of deformation in the Poisson effect.

## Results and Discussion

### Experiment 1

**Figure 2:**
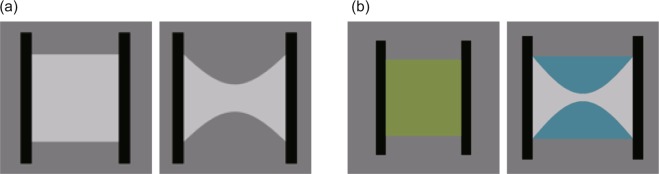
(**a**) Two snapshots of stimuli as used in Experiment 1. Left: the 1st video frame. Right: the 30th video frame. (**b**) The area which was used for the calculation of area ratio. The area ratio was calculated by dividing the blue area in the right panel by the green area in the left panel.

The purpose of this experiment was to explore whether Poisson’s ratio and/or the area ratio were the determinant of causality perception for orthogonal deformations in the Poisson effect. Two parameters (Fig. 2a) were manipulated: one was the magnitude of vertical compression (*d*_*y*_ in Formula 1), the other was the initial width of the material (*w* in Formula 1). As is clear from Formula 1, as *d*_*y*_ and/or *w* increase, Poisson’s ratio *ν* increases. The variation of the perception of causality with *ν* was examined. In addition, the relationship between the perception of causality and image parameters related to deformation magnitudes was examined.

First the experimental data were analyzed in terms of the two psychophysical parameters: the magnitude of vertical compressions and initial width. Second, the relationship between the psychophysical results and Poisson’s ratio was evaluated. Third, the relationship between the area ratio as an index of a deformation magnitude was examined. The experiment was controlled by using PsychoPy v1.83^16,17^.

**Figure 3:**
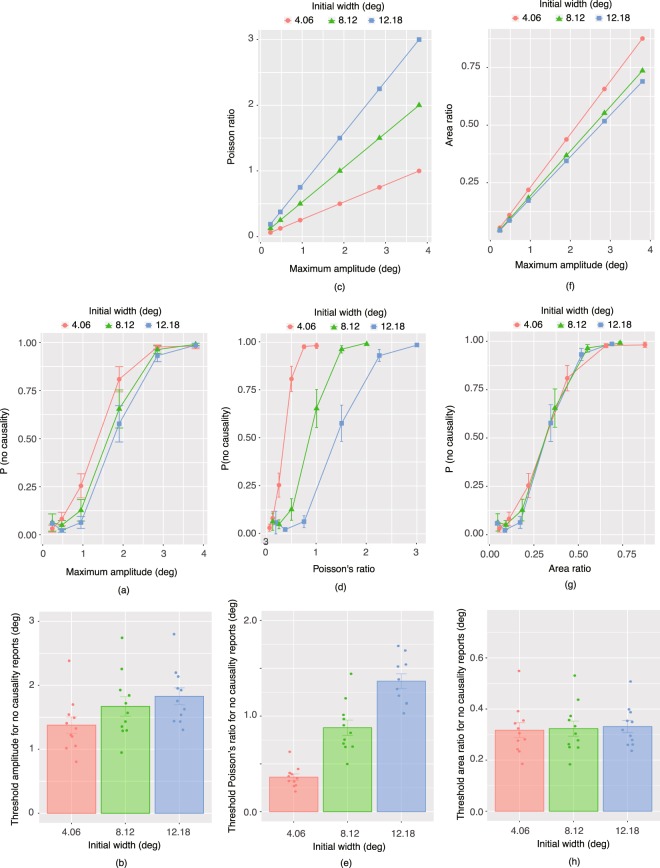
(**a**) Proportions of trials with no report of causality as a function of the maximum amplitude of vertical compressions. Error bars denote standard error of the mean (N = 11). (**b**) Threshold amplitude to cause a 50% proportion of reporting “no causality” for each of the initial width conditions. (**c**) Variations in Poisson’s ratio as functions of the maximum amplitude of vertical compressions and the initial width of materials. (**d**) The proportion of trials with no causality reported as a function of Poisson’s ratio. (**e**) Threshold Poisson’s ratio to cause a 50% proportion of reporting “no causality” for each of the initial width conditions. (**f**) Variations of the area ratio as functions of the maximum amplitude of vertical compressions and the initial width of materials. (**g**) The proportion of trials with no report of causality as a function of the area ratio. (**h**) Threshold area ratio to cause a 50% proportion of reporting “no causality” for each of the initial width conditions.

Figure 3a shows the mean proportion of trials wherein the observers reported ‘NO’ causal relationship between the horizontal extension and the vertical compression. First, using quickpsy^18^, a psychometric function was fitted to each individual’s data for each initial width condition, and the amplitude threshold required to cause a 50% rate of reporting “no causality” was calculated, as shown in Fig. 3b. Using a linear mix effects model, a one-way repeated measures ANOVA with the initial widths as a within-subject factor was calculated, which showed that the main effect of the initial widths was significant [*F*(2,20) = 31.78, *p* < 0.0001, *r*^2^ = 0.95]. Multiple comparisons after adjustment with the Holm’s method showed that the 4.06 deg condition was significantly different from the 8.12 deg condition (*z* = 5.11, *p* < 0.0001) and 12.08 deg condition (*z* = 7.85, *p* < 0.0001). The difference between the 8.12 deg and 12.08 deg conditions was also significant (*z* = *2*.745, *p* = 0.0061).

Figure 3c shows how Poisson’s ratio varied with the stimulus manipulation of initial widths and the magnitude of vertical compressions. Poisson’s ratio increased with the initial width as well as the magnitude of vertical compressions. The proportion of trials with no report of causality was plotted as a function of Poisson’s ratio in Fig. 3d. The results showed that the proportions did not consistently vary with Poisson’s ratio. In other words, the proportions were strongly dependent on the initial widths. Psychometric functions were again fitted to the proportions as a function of the Poisson’s ratio and the threshold Poisson’s ratio was calculated for each initial width condition (Fig. 3e). Using a linear mixed effects model, a one-way repeated measures ANOVA was calculated, with the initial widths as a within-subject factor, which showed that the main effect of the initial widths was significant [*F*(2,20) = 173.61, *p* < 0.0001, *r*^*2*^ = 0.95]. Multiple comparison tests after adjustment with the Holm’s method showed that the 4.06 deg condition was significantly different from the 8.12 deg condition (*z* = 8.91, *p* < 0.0001) and 12.08 deg condition (*z* = 18.62, *p* < 0.0001). The difference between the 8.12 deg and 12.08 deg conditions was also significant (*z* = 9.72, *p* = 0.0061). If human observers used Poisson’s ratio to determine apparent causality in the Poisson effect, no difference should have been observed among the initial width conditions because it would be expected that the cognitive system calculated the Poisson’s ratio on the basis of the initial width and the magnitude of vertical compressions. Based on these observations, it can be concluded that the observers did not assess causality between extension and compression deformations on the basis of Poisson’s ratio.

It may be necessary to clarify the relationship between Poisson’s ratio and initial widths. As described in Formula 1, the calculation of Poisson’s ratio uses the following two parameters: compression amplitude and initial width. If the cognitive system internally calculated Poisson’s ratio, the calculation would have been based on these two parameters. Please note here that no interaction could be assumed between Poisson’s ratio and the initial width, because the Poisson ratio itself is dependent on the initial width. Hence, it was predicted that no difference should have been observed among the initial width conditions if human observers used an internalized Poisson’s ratio, whose calculation used initial widths as a parameter. However, the results of Experiment 1 (Fig. 3d) showed that even when the Poisson’s ratio was 0.5, which could occur in the natural world, the proportion of no causality reports was strongly dependent on the initial widths. Moreover, the threshold Poisson ratio was statistically different among the initial width conditions. Taken together, the conclusion was that human observers do not use Poisson’s ratio to evaluate the causality of the perceived deformation.

Next, based on previous studies showing that image features do predict the perception of material properties^13–15^, the question became whether the area ratio was used to assess causality in the Poisson effect. Figure 3f shows how the area ratio varied with the magnitude of vertical compressions and the initial width of materials. Comparing Fig. 3a,f, the reader will notice that the pattern of variation was similar between the proportion of trials with no report of causality and the area ratio. Figure 3g shows a plot of the proportion of trials with no reported causality as a function of the area ratio, and shows that the proportion systematically varied with the area ratio. Importantly, the pattern of variations was similar among the three initial width conditions. The result of again fitting psychometric functions to the proportions as a function of the area ratio and calculating the threshold area ratio is shown in Fig. 3h. Using a linear mixed effects model and conducting a one-way repeated measures ANOVA with the initial widths as a within-subject factor showed that the main effect of the initial widths was significant [*F*(2,20) = 0.755, *p* = 0.48, *r*^*2*^ = 0.94]. The results indicate that irrespective of the initial width, human observers use similar area ratio magnitudes to assess causality in the Poisson effect.

### Experiment 2

**Figure 4:**
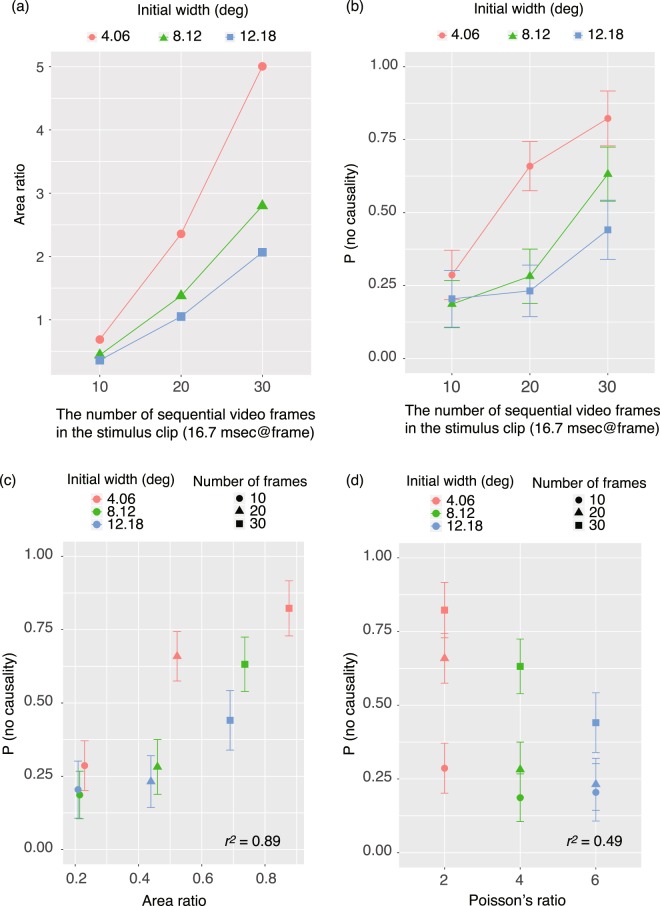
(**a**) Area ratio plotted as a function of the number of sequential video frames contained in the stimulus clip as used in Experiment 2. (**b**) Proportion of trials with no report of causality as a function of the number of sequential video frames contained in the stimulus clip. Error bars denote ± 1 standard errors of the mean (N = 11). (**c**) Correlation plots between the area ratio and the proportion of trials with no report of causality. (**d**) Correlation plots between Poisson’s ratio and the proportion of trials with no report of causality.

The purpose of this experiment was to confirm whether the area ratio was a determinant of the assessment of causality of deformations in the Poisson effect. Even with keep both the maximum amplitude of vertical deformation and initial width of a material constant, the area ratio depends on the number of sequential video frames (Fig. 4a). That is, as the number of sequential video frames increases, the area ratio linearly increases. Importantly, Poisson’s ratio does not vary with the number of sequential video frames. If the assessment of causality of deformations in the Poisson effect was dependent on the area ratio, the proportions of trials with no assessment of causality would be dependent on the area ratio that was modulated by the number of the sequential video frame.

Figure 4b shows the proportion of trials with no report of causality, as a function of the number of sequential video frames contained in the stimulus clip. There was a clear tendency for the proportion to increase with the number of sequential video frames. In addition, the proportion decreased with the initial width of the material, consistent with the results of Experiment 1. I conducted a two-way repeated measures ANOVA of the proportion, with the number of video frames and initial widths as within-subject factors. The main effect of the number of video frames was significant [*F*(2,20) = 4.95, *p* = 0.0179, *η*_p_^2^ = 0.33]. Multiple comparison tests using a Bonferroni correction showed that the proportion in the 10-frame condition was not significantly different from the proportion in the 20-frame condition (*p* = 0.13), but was significantly different from the proportion in the 30-frame condition (*p* < 0.001). The proportion in the 20-frame condition was significantly different from that in the 30-frame condition (p < 0.0001). The main effect of the initial widths was significant [*F*(2,20) = 21.82, *p* < 0.0001, *η*_p_^2^ = 0.69]. Multiple comparison tests using a Bonferroni correction showed that the proportion in the 4.06 deg condition was significantly different from both the proportion in the 8.12 condition (*p* < 0.0001) and the proportion in the 12.18 deg condition (*p* *<* 0.0001). The proportion in the 8.12 condition was significantly different from the proportion in the 12.18 deg condition (*p* = 0.031). Interaction between the two factors was also significant [*F*(4,40) = 11.92, *p* < 0.0001, *η*_p_^2^ = 0.54]. The value of *r*^*2*^ was also calculated for a linear fitting function between the proportion of trials with no report of causality and the area ratio (Fig. 4c); and between the proportion of trials with no report of causality and Poisson’s ratio (Fig. 4d). It was found that the proportion was correlated more strongly with the area ratio (*r*^*2*^ = 0.89) than with Poisson’s ratio (*r*^*2*^ = 0.49).

The results indicate that, consistent with Experiment 1, the proportion of trials with no reported causality depended on the area ratio, that is, on the ratio of the area of deformed regions to the area of original material regions (Fig. 4c). The results support the preliminary idea obtained in Experiment 1—that observers make use of image features related to the magnitude of deformation to assess the causality of deformations in the Poisson effect. Consistent with Experiment 1, Poisson’s ratio could not satisfactorily account for the proportion of trials with no reported causality. The results suggest that human observers do not directly represent the physical characteristics of elastic materials, at least in order to calculate the causal relationship between extension and compression deformations in the Poisson effect.

Since the results of Experiment 1 clearly showed that human observers do not use Poisson’s ratio to judge the causality via the Poisson effect, the role of Poisson’s ratio on the apparent causality in the experimental design was not closely checked. Actually, the results of Experiment 2 showed that the proportion of “no causality” reports was negatively correlated with the Poisson’s ratio, when a positive correlation should have been observed if Poisson’s ratio was critical. The results again suggest that Poisson’s ratio is not an explanatory factor for the causality perception.

Still, it is necessary to discuss why negative correlation was observed between Poisson’s ratio and the proportion of no causality reports. In the stimuli of Experiment 2, the initial width was a strong modulatory factor in Poisson’s ratio. Specifically, when the initial widths were 4.06, 8.12, and 12.18 deg, the Poisson’s ratio was approximately 2.0, 4.0, and 6.0, respectively. Thus, there was a possibility that the proportions were negatively correlated with the initial widths, not in agreement with Poisson’s ratio. In Experiment 1, the larger initial widths caused the lower proportions of “no causality” reports, which was quite consistent with the results of the current experiment. Based on these results, it was concluded that the negative correlation between Poisson’s ratio and the proportion of “no causality” reports was a spurious correlation and that the actual correlation occurred between the initial widths and the proportion of “no causality” reports.

### Experiment 3

**Figure 5:**
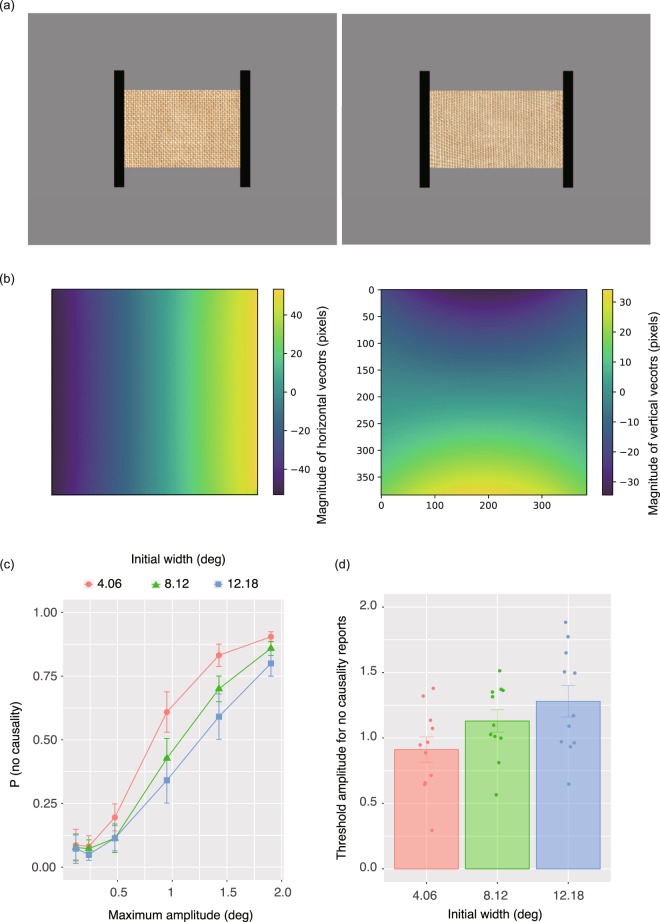
(**a**) Snapshots of a stimulus clip used in Experiment 3. Left and right panels show the initial (the 1st) and final (the 30th) video frames, respectively. (**b**) Optical flow fields of the simulation video clip (Fig. 1). Left and right panels respectively show horizontal and vertical motion vectors between the 1st and the 30th frames of the simulation video clip. (**c**,**d**) Experiment 3 results. (**c**) Proportion of trials with no causality report as a function of maximum amplitude. Error bars denote ± 1 standard errors of mean (N = 11). (**d**) Threshold amplitude to cause 50% proportion of no report of causality for each initial width condition. Error bars denote ± 1 standard errors of the mean (N = 11).

The purpose of this experiment was to check whether the assessment of causality by observers relies on the contour shape of the material in the final frame of the video clip. As shown in Figs 1, 2a, in the stimulus clips used in the previous experiments, the shape of the material changed greatly before and after deformation in the Poisson effect. There was thus the possibility that observers compared the static shape of a material before and after the deformation, and reported no causality if the deformation magnitudes were too large for an internal criterion for assessment of causality. Another possibility was that observers used dynamic aspects of deformations in order to assess causality. Specifically, instead of comparing static snapshots of a material before and after deformation, the observers might use dynamic deformation cues to assess deformation magnitudes as a cue to causality of deformation in the Poisson effect. To assess this possibility, it was necessary to rule out visual cues that were relevant to shape comparisons before and after deformations. In this experiment, we eliminated shape cues from the clip. Textured material surfaces were used (Fig. 5a) to add dynamic texture changes during the deformation. Here, the outer contour of a deforming shape was not visible; only the central region of the surface was visible, seen through a rectangular aperture the shape of which was extended in accordance with the movement of two black bars, as used in the stimuli of the previous experiments. The observers’ assessment of causality would be dependent on both the initial width and the maximum amplitude, in a similar way to the pattern observed in Experiment 1, provided that the observers assessed causality using dynamic image deformation in the video clip.

Figure 5c shows the proportion of trials with no causality report as a function of the amplitude of vertical compression. As in Experiment 1, a psychometric function was fitted to the individual data of each initial width condition, and calculated the amplitude threshold required for the 50% “no report of causality” in Fig. 5d. As in Experiment 1, using a linear mixed effects model, a one-way repeated measures ANOVA was conducted with the initial widths as a within-subject factor, which showed that the main effect of the initial widths was significant [*F*(2,20) = 12.551, *p* < 0.0002, *r*^*2*^ = 0.85]. Multiple comparisons after adjustment with the Holm’s method showed that the 4.06 deg condition was significantly different from the 8.12 deg condition (*z* = *2*.95, *p* < 0.007) and 12.08 deg condition (*z* = 4.982, *p* < 0.0001). The difference between the 8.12 deg and 12.08 deg conditions was also significant (*z* = 2.031, *p* < 0.05).

The results indicate that human observers are able to assess the causality of deformations in the Poisson effect without comparing the shape of material before and after deformation. This suggests that human observers can use motion patterns to infer the causality of deformation, consistent with previous studies proposing that image motion is a strong cue in inferring material properties^1,4–6^. On the other hand, the results do not necessarily tell us that the human visual system does not use shape cues to assess the causality of deformation in the Poisson effect. Rather, the results indicate that the observers could assess causality without the aid of explicit shape differences before and after deformation. Because we simply eliminated shape cues from stimuli, it is still unclear how shape and motion cues interact with each other, and this may be worth checking in future studies.

In the 30th frame, texture patterns on the material surface were strongly deformed, so one might argue that the comparison of the magnitude of texture deformations served as a cue to the assessment of causality. This possibility has not been empirically ruled out in this experiment. However, though rather speculative, the possibly is unlikely. When we examine the right panel of Fig. 5a, we do not get the impression that the material in the panel is laterally extended, but when we view the clip, the impression is fairly compelling. Thus, it is unlikely that observers could utilize the static deformation as a cue to material extension as well as compression. The critical phenomenal difference between static and dynamic presentation of stimuli indicates that observers predominantly used dynamic deformation cues to infer causality of deformation in the Poisson effect, when shape information was absent.

## General Discussion

The findings suggest that instead of using Poisson’s ratio, the observers employed image cues (the area ratio) related to deformation magnitudes in order to assess the causality of deformation in the Poisson effect (Experiments 1 and 2). Shape comparisons before and after deformations were not always necessary for the assessment of causality of deformation (Experiment 3).

Although previous studies have reported that human observers can perceive a force in the so-called launching effect and its variants^19,20^, the present study first shed light on the matter of how human observers perceive the causality of deformations in an elastic object. There are many physical events that are related to force perception, but few examples have been tested from a psychological perspective. The approach in the present study may represent a promising direction for investigating how the perception of force is linked to the perception of materials.

From the perspective of motion perception, an interesting issue is how the human visual system perceives a material that is horizontally extended and vertical compressed. Previous studies have shown that motion vectors are averaged^21,22^ or integrated^23^ into perception of a single coherent direction of motion. In addition, different studies have also reported that human observers could integrate local motion signals into a complex motion pattern such as dilation, divergence, shear, and vorticity^24–26^. Among the vector patterns, our stimuli contained a shear flow pattern. The visual system perhaps tries to detect a shear pattern and evaluate whether it comes from the extension/compression deformations of a single material. Another possibility is that the visual system decomposes orthogonal motion vectors^27^ and groups the vectors into a hierarchical structure within a single material^28^. When the combination of orthogonal motion vectors does not meet a single criterion required to create a hierarchical structure, the impression of causality between the orthogonal motion vectors in our stimuli may be broken.

It is necessary to carefully discuss whether human observers do not actually represent physical properties of material. In this study, although both physically expected and unexpected versions of the Poisson effect were presented, Poisson’s ratio was an explanatory factor in neither the range of physically expected nor unexpected Poisson effects (Figs 3d and 4d). Although it was clear that Poisson’s ratio was not used for the determination of causality perception in the Poisson effect, it is necessary to carefully consider whether or not Poisson’s ratio is used by the visual system at all. It is still possible that the area ratio is used for the crude analysis of whether deformations in the Poisson effect are causally related or not; after causality is internally established, the visual system might use Poisson’s ratio to assess the finer details of the physical aspects of the material.

Some stimuli in this study contained sinusoidal vertical compressions which might possibly be interpreted as shape changes due to depth. Specifically, in some stimuli with large vertical compressions, some observers may have perceived a material as if it were being pulled in a receding direction. On the other hand, the interpretation of shape change in depth may arise only when the interpretation of the Poisson effect is unlikely. That is, a shape change in depth may be perceived only when human observers interpret that the source of a vertical deformation cannot be attributed to a horizontal extension alone. It would be intriguing to investigate the relationship between the brain’s attribution of the source of image deformation and deformation features in stimuli.

It would be essential to discuss the relationship between the causality judgment of deformations in the Poisson effect and material perception. In a preliminary pilot experiment some observers were asked to freely report the material types they saw during the observation of stimulus clips. Elastic materials were consistently reported when the amplitude was small, while reported material types were not consistent among the observers when the amplitude was large. More importantly, in debriefing they reported determining the perceived type of material depending on the assumed causal relationship between horizontal and vertical compressions. The debriefing outcome led to the supposition that causality perception might precede material perception. That is, when the vertical compression magnitude was large, the observers could not find the causal relationship between the horizontal and vertical deformations, and likely reported inconsistent material types. This set of observations indicates that the observers may rely on their judgment of material types in the presence/absence of causality and that the causality judgment is not based on material perception. On the other hand, it is dangerous to make a hasty conclusion that the causality judgment of deformation always precedes material perception. It may depend on the types of image deformation and/or material types in stimulus clips. Careful examination in separate studies will be necessary to determine the relationship between causality perception and material perception.

## Method

### Experiment 1

*Observers* Eleven naive people (seven females and five males) participated in the experiment. Their mean age was 35.5 (SD10.4). All observers in this study reported having normal or corrected-to-normal visual acuity. They were recruited from outside the laboratory and received payment for their participation. Ethical approval for this study was obtained from the ethics committee at Nippon Telegraph and Telephone Corporation (Approval number: H28-008 by NTT Communication Science Laboratories Ethical Committee). The experiments were conducted according to principles that have their origin in the Helsinki Declaration. Written, informed consent was obtained from all observers in this study.

*Apparatus* Stimuli were presented on a 21-inch iMac (Apple Inc. USA) with a resolution of 2048 × 1152 pixels and a refresh rate of 60 Hz. A colorimeter (Bm-5A, Topcon, Japan) was used to measure the luminance emitted from the display. A computer (iMac, Apple Inc., USA) controlled stimulus presentation, and data were collected with PsychoPy v1.83^16,17^.

*Stimuli* were video clips that consisted of white material with a rectangular shape, two black bars that pulled the material horizontally, and a gray background (Fig. 2a and Supplementary clip 1). The luminance of the material was 51 cd/m^2^. At the first video frame of the stimulus video clip, the vertical height of the material was 8.12 deg. The height gradually deformed so that the top and bottom sides of the material underwent a spatially sinusoidal modulation. The maximum amplitude of the modulation was randomly chosen from one of the following six levels: 0.24, 0.48. 0.96, 1.90, 2.86, and 3.80 deg. The top and bottom sides of the materials linearly deformed across a video clip consisting of 30 video frames. At the first video frame of the stimulus video clip, the horizontal width of the material was randomly chosen from one of the following three levels: 4.06, 8.12, and 12.18 deg. The width was linearly extended so that the maximum width reached 5.96, 10.02, and 14.08 deg at the 30th frame of the stimulus clip. That is, with all of the width conditions, the width extended 1.902 deg in the end. The height and width of the black bars were 12.2 and 1.0 deg, respectively. The luminance of the bars was 0.2 cd/m^2^. In the stimulus clip, the black bars moved linearly by 1.902 deg so that the right and left edges of the left and right black bars were respectively attached to the left and right edges of the white material. The horizontal level of the material was matched with the horizontal level of the black bar. The material and bar were presented against a gray background with a luminance of 20 cd/m^2^.

*Procedure* Each observer was individually tested in a dimly lit chamber. The observers sat 64 cm from the display. With each trial, the first video frame of the stimulus clip was displayed for 0.5 sec, followed by the presentation of the stimulus clip for 0.5 sec (30 video frames in total). Finally, immediately after playing the clip, the final (30th) video frame of the stimulus clip was displayed for 0.5 sec. After the disappearance of the stimulus clip, the observer assessed whether the horizontal extension of the material was causally related to the vertical compression of the material in a binary, forced-choice manner. Observers were explicitly told that the causality of deformation in stimulus clips meant that the vertical compression in the material was mechanically caused by the horizontal extensions in a physically plausible manner. The observers reported their assessment by pressing one of two assigned keys. Each observer had four sessions, each consisting of 3 initial widths × 6 compression amplitudes × 5 repetitions. Within each session the order of trials was pseudo-randomized. Thus, each observer had 360 trials in total. It took 30–40 minutes for each observer to complete all of four sessions.

### Experiment 2

*Observers* Eleven naive people (seven females and four males), who had not participated in Experiment 1, participated in this experiment. Their mean age was 34.6 (SD 9.6).

*Apparatus* The apparatus was identical to that as used in Experiment 1.

*Stimuli* These were also identical to those as used in Experiment 1 except for the following. In this experiment, the number of video frames contained in the stimulus clip was manipulated. A stimulus clip was presented for 0.167 sec (from the 1st to the 10th frames), 0.334 sec (from the 1st to the 20th frames), and 0.5 sec (from the 1st to the 30th frames). All six maximum amplitude conditions as used in Experiment 1 were no longer tested. The focus here was on the 3.80 deg condition, which was the greatest of the maximum amplitude conditions used in Experiment 1.

*Procedure* As in Experiment 1, each observer was individually tested in a dimly lit chamber. The observers sat 64 cm from the display. For each trial, the first video frame of the stimulus clip was displayed for 0.5 sec, followed by presentation of the stimulus clip for 0.167 sec, 0.334 sec, or 0.5 sec (10, 20, and 30 video frames in total). Finally, immediately after playing the clip, the last (10th, 20, and 30th, respectively) video frame of the stimulus clip was displayed for 0.5 sec. After the stimulus clip disappeared, the observer assessed whether the horizontal extension of the material was causally related to the vertical compression of the material in a binary, forced-choice manner. The observer reported his/her assessment by pressing one of two assigned keys. Each observer participated in two sessions, each consisting of 3 initial widths × 3 different numbers of video frames × 10 repetitions. Within each session the order of trials was pseudo-randomized. Thus, each observer underwent 180 trials in total. It took 15–20 minutes for each observer to complete both sessions.

### Experiment 3

*Observers* Eleven naive people (seven females and five males), who had participated in Experiment 1 participated in the experiment. They were still naive as to the purpose of the experiment because no debriefing about the purpose of the experiment was provided to them after Experiment 1.

*Apparatus* The apparatus was identical to that as used in Experiment 1.

*Stimuli* These were also identical to those used in Experiment 1, apart from the following changes. Instead of white material, a material with a textured surface was used, as shown in Fig. 5a (See also Supplementary clip 2). As the black bars moved laterally, textures inside the material also deformed so that the textures were horizontally elongated and vertically compressed. For vertical compression, the deformation was spatially modulated in a sinusoidal manner. Analysis of the optical flow fields of the video clip for an extended cloth is shown in Fig. 1, showing that the optical flow fields had a linear pattern across both horizontal and vertical dimensions (Fig. 5b). Therefore, similar patterns of linear changes in deformation fields were applied to the deformation of the texture patterns in the stimuli. As in Experiment 1, three levels of initial widths of the material were tested: 4.06, 8.12, and 12.18 deg. Here, the following six levels of maximum amplitude modulation were used: 0.12, 0.24. 0.48, 0.96, 1.43, and 1.90 deg. The amplitudes were just half of those used in Experiment 1. The reason for halving the values was the experimental failure to correctly control the spatial deformation magnitude based on the original maximum deformation amplitude. In the video clip, the contour of the material was not visible. The large size of the textured surface was deformed on the basis of the parameters described above, and the central region of the textured surface was clipped so that the vertical height was kept constant at 8.12 deg, varying the horizontal width in accordance with the movement of the two black bars in a way similar to that used in Experiment 1. Deformation was completed across 30 video frames as in the stimuli of Experiment 1.

*Procedure* This too was identical to that used in Experiment 1.

## Supplementary information

Video 1

Video 2
